# Effect of Curing Parameter on the Performance of Electric-Induced Heating-Cured Carbon Fiber-Reinforced Conductive Cement-Based Materials: Experiment and Finite Element Method Analysis

**DOI:** 10.3390/ma18174057

**Published:** 2025-08-29

**Authors:** Jiabin Xie, Yishu Zhang, Weichen Tian, Zhanlin Zhang, Wei Wang

**Affiliations:** 1 The School of Civil Engineering, Harbin University, Harbin 150030, China; 2 School of Civil Engineering, Harbin Institute of Technology, Harbin 150030, China; 3 School of Infrastructure Engineering, Nanchang University, Nanchang 330031, China

**Keywords:** fiber-reinforced concrete, carbon fiber, electric resistance, curing temperature

## Abstract

Winter concrete construction is a pivotal engineering issue that needs to be addressed due to the failure of cementitious materials to hydrate under severely low temperatures. To solve the problem, the electric-induced heating curing (EIH) method was presented to prepare cement mortar (CF-CM) at an environmental temperature of −20 °C. The influence of some key parameters, including carbon fiber (CF) content (0–0.9 vol%), preparation methods, and EIH curing regimes (constant power vs. constant voltage; frequency: 30–70 Hz), on the performance of CF-CM were examined. Furthermore, the curing temperature of EIH-cured specimens were simulated based on COMSOL Multiphysics software. The results demonstrated that the electrical percolation threshold of CFs inside the specimen was 0.6 vol%. EIH curing achieved 1-day early strength equivalent to 2 days of standard curing, and increasing CF content showed little influence on the mechanical properties of CF-CM specimens. Moreover, constant-power EIH maintained stable curing temperatures (>50 °C), outperforming unstable constant voltage curing. Applied frequency (30–70 Hz) exhibited negligible impact on compressive strength, validating standard 50 Hz AC for practical application. Furthermore, the optimal EIH power density identified based on COMSOL Multiphysics software was 667 W/m^2^, successfully maintaining specimen temperatures between 60 °C and 70 °C to enable rapid strength development under sub-zero conditions, laying a foundation for the use of COMSOL in the guidance of EIH curing regime design. This work provides a scientifically grounded and applicable solution for winter concrete construction.

## 1. Introduction

Concrete, as the cornerstone of modern infrastructure, faces a persistent challenge in cold climates: the severe retardation or complete cessation of hydration reactions at sub-zero temperatures [1,2]. This drawback significantly delays strength development, prolongs construction schedules, increases vulnerability to early-age frost damage, and finally compromises the long-term durability and structural integrity of concrete structure fabricated in cold regions under low temperatures [3,4,5]. It should be noted that traditional concrete curing methods for winter construction, such as the use of insulating blankets, heated enclosures, or chemical accelerators, show substantial drawbacks. These methods are often energy intensive, logistically complex, costly, environmentally taxing, and sometimes insufficient for achieving the rapid, high early-strength gains demanded by accelerated construction timelines or repair scenarios in harsh environments [6,7,8]. Consequently, the development of innovative, efficient, and reliable curing techniques capable of effectively promoting cement hydration and strength development under negative-temperature conditions remains a critical research frontier.

Among the novel curing methods, electric-induced heating (EIH) curing is a promising method utilizing the intrinsic or enhanced electrical conductivity of the cementitious matrix itself [9,10,11] by directly passing an electrical current through the material, aiming to realize the rapid and uniform temperature improvement [12,13,14]. Moreover, the EIH method demonstrates advantages in sub-zero environments compared to conventional surface-heating approaches. Its volumetric nature ensures more efficient and homogeneous heat distribution within the specimen, thus minimizing thermal gradients and the associated risks of cracking [15]. Moreover, the improved curing temperature can stimulate the hydration reaction inside the sample, which can effectively accelerate the strength development even at temperatures below freezing, potentially revolutionizing winter concreting and enabling rapid repairs in cold regions. However, it should be noted that the effects of some basic curing parameters on the performance development of EIH-cured specimens were still not elucidated.

However, it should be noted that the successful implementation of EIH curing relies on the excellent electric conductivity of the sample [16,17]. While plain cement paste or mortar possesses some ionic conductivity, it is generally too low for efficient and practical EIH curing at viable voltages [18,19,20,21]. This necessitates the strategic incorporation of conductive fillers to form percolating conductive networks that can effectively reduce electric resistance. Among the selection of potential conductive additives, including carbon black, graphite, steel fibers, and various nanomaterials, carbon fiber (CF) stands out as an exceptionally suitable candidate for enhancing cementitious composites intended for EIH curing [22,23,24,25]. Tian et al. pointed out that the inclusion of CFs effectively enhanced the basic electric conduction inside cement concrete [26,27], and Liu et al. reported the beneficial effects of EIH curing on promoting the strength formation of high-performance concrete in comparable curing duration with traditional steam curing method [28,29]. CFs show exceptional electric conductivity with high toughness, enabling the efficient EIH curing process with the ability to improve the mechanical properties of cement-based materials. Moreover, CFs possess superior corrosion resistance compared to metallic alternatives, ensuring long-term stability within the highly alkaline cement environment without detrimental oxidation or rusting, which can degrade both electrical and mechanical performance [30,31,32,33]. Furthermore, CFs demonstrate outstanding thermal stability and high thermal conductivity, facilitating efficient heat transfer within the sample during the EIH curing process [34,35,36]. Consequently, carbon fiber is selected in this study as the optimal conductive filler to enable and enhance the EIH curing process in cement-based materials.

Furthermore, although EIH showed great potential on improving the performance of cement-based materials under negative temperature, its practical effectiveness is not guaranteed and hinges critically on the precise optimization of several key processing parameters. To be specific, the efficiency of the EIH curing process is profoundly influenced by specific operational variables including CF contents and electric field parameters. As for the CF contents, it is clear that the low content of CFs fails to establish the necessary percolation network for effective and uniform EIH curing, while excessive amounts can detrimentally impact workability, strength development, and cost. Moreover, selecting the optimal frequency range is also essential for maximizing heat generation efficiency within the material while minimizing energy loss and potential adverse effects on the microstructure. Furthermore, a significant challenge lies in the precise control and monitoring of the thermal conditions within the sample during EIH application. Surface measurements often fail to capture internal thermal gradients, and the establishment of a robust finite element model or other predictive computational model capable of accurately simulating the complex heat generation and transfer phenomena within the EIH cured sample is necessary.

To address the above issues, this study presents a comprehensive investigation into the development and optimization of carbon fiber-reinforced cement mortar (CF-CM) for efficient EIH curing at −20 °C. The percolation threshold of CFs in cement mortar was experimentally determined, and the effects of fiber dosage, preparation method, and curing regime on the early-age mechanical properties were tested. Moreover, COMSOL Multiphysics^®^ (Version 6.1) simulations were employed to model the coupled electro-thermal processes during EIH curing of CF-CM specimens cured at −20 °C, aiming to characterize the temperature development regularity and ultimately identify the electrical power input required to achieve the required curing temperature. This work aims to establish a robust foundation for the practical application of EIH curing as a rapid and reliable curing technology for cement-based materials in extreme cold environments.

## 2. Materials and Methods

### 2.1. Raw Materials

P.O. 42.5 cement was used as the main supplementary material, and the chemical com and mineralogical compositions of the cement have been added in Table 1 and Table 2. ISO standard sand was selected as the fine aggregate. The fineness modulus was medium sand in the medium zone, and the particle size distribution was 3.0 in the medium zone. The SiO_2_ content in the ISO standard sand was greater than 96%, which belongs to natural quartz sea sand, and the specific heat capacity of the sand was calculated of 0.92 kJ/(kg·K) [37]. The conductive filler used in this experiment was 4 mm chopped carbon fiber produced by Toray Corporation of Japan, with a carbon content of more than 95%, a diameter of 7 μm, a density of 1.76 g/cm^3^, a ductility of 1.5%, a tensile modulus of 220 GPa, and a tensile strength of 3800 MPa. The volume resistance was measured to be 1.5 × 10^−3^ Ω·cm. The water-reducing agent used in this experiment was a polycarboxylate high-efficiency water-reducing agent.

### 2.2. Mix Proportion

The basic mix proportion design of the CF-CM sample is detailed as below: The water-to-cement ratio was 0.35, and the sand-to-cement ratio was 1.5; the CF contents varied from 0 vol% to 0.9 vol% to determine the percolation threshold of CFs to form a fully connected conductive path in cement-based materials, and the initial resistance of the fresh specimen was used as the judgment criterion.

### 2.3. Preparation Methods

Due to the agglomeration of CFs, it is difficult to achieve a good dispersion effect. Therefore, in this experiment, CFs and cementitious materials were added simultaneously in the Hobart mixer and then mixed with the mixing water for stirring. Specifically, four different preparation methods were respectively adopted to prepare CF-CM, as shown in Table 3. Different preparation processes mainly studied the influence of sand addition sequence and stirring speed on the early strength of CF-CM.

### 2.4. Curing Regime

The samples cured by EIH curing was immediately transferred into the freezer with an environmental temperature of −20 °C; the voltage regulator was used as the power supply, and the curing duration for EIH was set to be 1 day. Collectively, the environmental temperature for EIH-cured specimen was −20 °C, with the internal humidity of freezer measured to be 75%.

The standard (SD) curing method was used as the comparison with the curing duration of 2 days, and the curing temperature of SD curing was 20 ± 2 °C, with the relative humidity above 95%.

In order to verify the influence laws of different EIH curing regimes on the properties of cement-based materials, two EIH curing regimes were compared: constant-power EIH curing and constant-voltage EIH curing. The curing age was set to be 12 h. The curing temperature of the specimens was measured to verify the effects of these two regimes. The specific design of the EIH curing method is as follows:

Constant-power EIH curing method: In the −20 °C negative-temperature condition, the CF-CM specimens were cured using a 30 W power supply, the resistance of the specimens was measured every 30 min, and the voltage of the specimens was adjusted in time based on Ohm’s law to ensure the implementation of the constant power regime.

Constant-voltage EIH method: The CF-CM specimens were cured under −20 °C using the constant voltage, which was determined at the initial curing stage based on the 30 W curing regime.

### 2.5. Test Methods

#### 2.5.1. Electric Resistance Test

**Figure 1:**
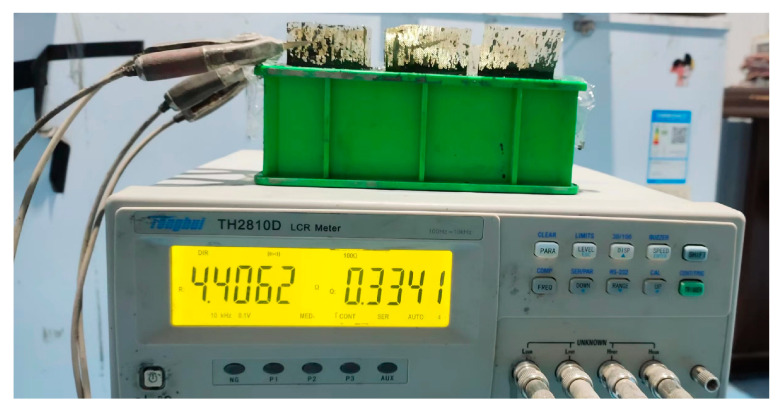
Illustration of electric resistance measurement.

The two-electrode method was used to measure the electric resistance of CF-CM specimens (see Figure 1) with a testing frequency of 10 kHz, which was reported to eliminate the interface resistance. For explanation, the unit of “4.4062” in Figure 1 was “Ω”.

#### 2.5.2. Mechanical Strength Test

The compressive strengths of CF-CM specimens cured by different curing conditions were tested. The specimen dimensions are all 50 mm × 50 mm × 50 mm. After curing to the corresponding age (EIH curing for 1 day and SD curing for 2 days), the compressive strength of the specimens was tested with a force loading rate of 2.4 ± 0.2 kN/s. Six specimens were prepared for the strength test, and the average value was used as the final result.

#### 2.5.3. Temperature Test

To measure the curing temperature of EIH-cured specimen, the thermocouple was inserted at the center of CF-CM specimen, and the JK32-channel temperature acquisition instrument (Jinko, Changzhou, China) was used to provide real-time feedback on the center temperature of the specimen during the EIH curing process.

### 2.6. Finite Element Modeling Analysis

**Figure 2:**
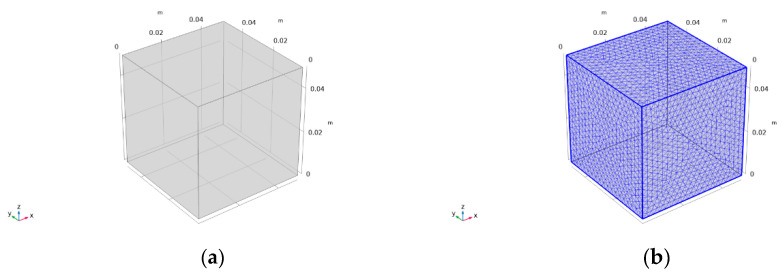
Model establishment of CF-CM specimen: (**a**) geometric model and (**b**) model element division.

In this experiment, the EIH curing process of the CF-CM specimens under a negative-temperature environment (−20 °C) is a non-steady-state heat transfer process. COMSOL simulation software was used, and relevant simulation calculations were conducted for the EIH curing process to clarify the development of the specimen temperature under different curing conditions. This can better predict the power supply and temperature development of the EIH-cured specimens from the perspective of numerical simulation. The specific steps of the numerical simulation are as follows:

(1) A geometric simulation model of the specimen was established, and the specimen model units were divided. According to the experimental design dimensions, the same model size of 50 mm × 50 mm × 50 mm was determined. As shown in Figure 2. at the same time, in order to obtain the internal temperature field of the specimen, 15 domain point probes were arranged, including the center point of the model (0.025, 0.025, and 0.025), to monitor the internal temperature changes of the specimen during the EIH curing process. To achieve more accurate results, a more refined grid division was used, with a complete grid containing 100,140 domain units, 4692 boundary units, and 216 edge units. The result of the model unit division is shown in Figure 2b.

(2) Setting the material-related parameters and modifying the values of thermal conductivity and specific heat capacity in the material library of the COMSOL software system: The specific heat capacity of the mortar specimens was calculated by the weighted average method of mass. The specific heat capacity of the mixing water was taken as 4.2 kJ/(kg·K), and the specific heat capacity of the mortar specimens was calculated to be 1.29 kJ/(kg·K) [37].

(3) Establishing the relevant physical fields: A Joule heat module that simultaneously includes the solid heat transfer module and the current module was selected. The distribution of electric field, current, and potential in the specimen (conductive medium) was calculated using the current module, and different heat exchange methods were added in the solid heat transfer module.

(4) Establishing boundary conditions and setting various user-defined parameters: In the current boundary condition, the potential of the left surface was set to 0, and the potential of the right surface was set to U. The calculation was carried out according to the actual power of the EIH-cured specimens. In the boundary conditions of solid heat transfer, the default physical properties of the mortar specimen model (specific heat capacity, thermal conductivity, resistance) did not change with the temperature. The environmental temperature of the specimen model was set to −20 °C (253.15 K), and the initial temperature of the mortar specimen was set to 20 °C (293.15 K).

(5) Calculating and outputting the results: During the transient process of EIH curing, the calculation step was set to 20 min, and the calculation time was 720 min. The temperature of each temperature detection point was calculated and output.

## 3. Results and Discussion

### 3.1. Effect of CFs on the Electrical Properties

**Figure 3:**
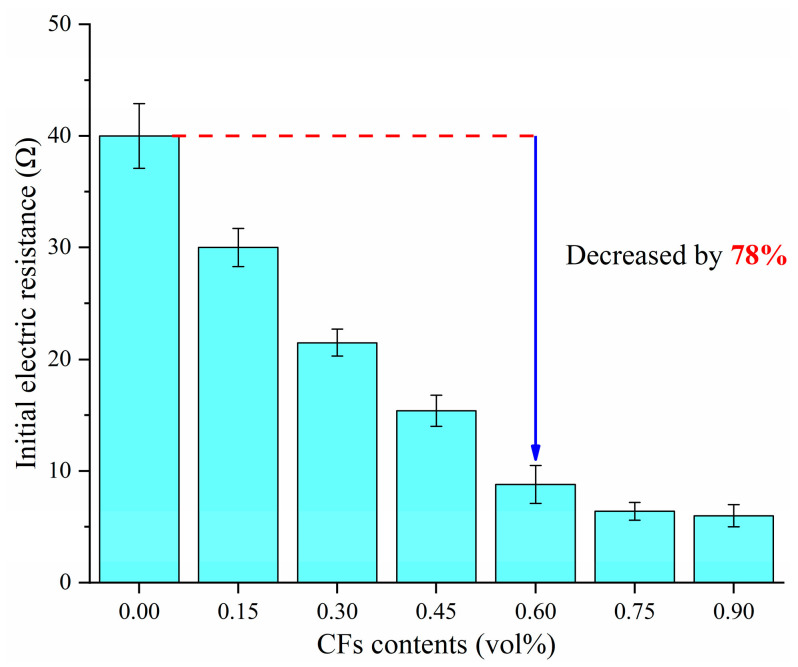
Electric resistance of CF-CM containing various CF contents.

The initial resistance of fresh specimens with different CF contents are shown in Figure 3, and the concept of initial resistance refers to the measured resistance of fresh specimen when it was just cast into the mold. It can be observed from the results that the resistance of the specimens decreased significantly with the increase in CF content. The variation of initial resistance with carbon fiber dosage can be divided into two stages. Without adding CFs, the resistance of the fresh mortar can reach 40 Ω, at which point the specimens mainly rely on the ionic conduction in the pore solution. When the CF dosage increased to 0.6 vol%, the initial resistance of the fresh mortar was 8.8 Ω. As the CF dosage continued to increase, the rate of decrease in resistance was significantly smaller compared to before. Based on the experimental results, it can be concluded that a stable conductive network was formed in the mortar specimens when the CF content was 0.6 vol%. Therefore, it is determined that the percolation threshold of CFs for forming a completely connected conductive path within the fresh cement mortar is 0.6 vol%.

### 3.2. Effect of Preparation Method on the Mechanical Properties

**Figure 4:**
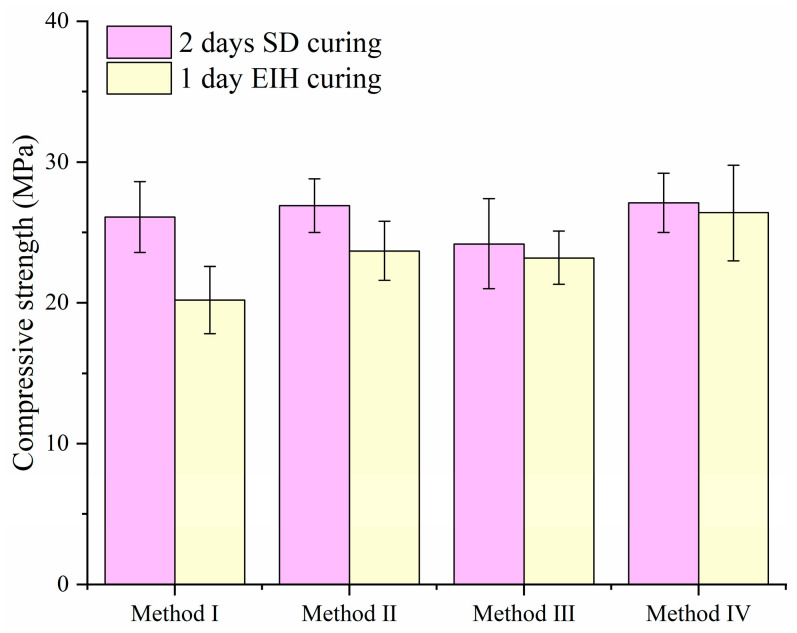
Compressive strength of CF-CM cured by different preparation methods.

In this section, the effect of the preparation methodology on the compressive strength development of CF-CM specimens was investigated, with the CF content constant at 0.9 vol%, as revealed in Figure 4. It can be noticed that the specimens fabricated using preparation method IV consistently demonstrated the highest compressive strength amongst the methods evaluated. This superior performance indicated that the drying mix stage played a fundamental role in determining the final mechanical properties. The observed strength enhancement is directly linked to the effectiveness of fiber dispersion within the cementitious matrix. The drying mix stage appears to be paramount for achieving a more uniform and effective distribution of the CFs throughout the specimen. Based on this result, the preparation method IV was used for the following experiments.

### 3.3. Effect of EIH Curing Regime and Electric Field Frequency

**Figure 5:**
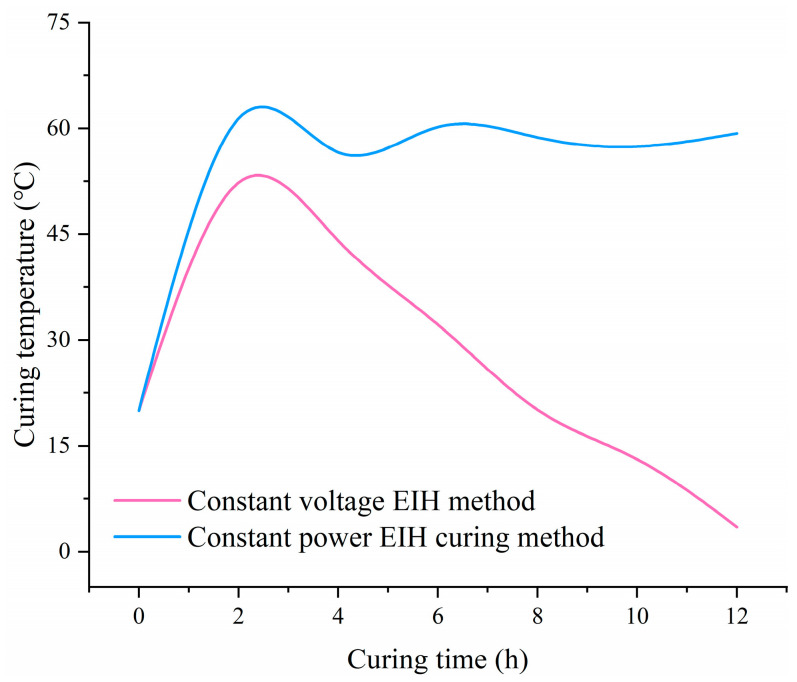
Curing temperature development of CF-CM specimens cured by different EIH regimes.

During the 12 h EIH curing period, CF-CM specimens were prepared in a sub-zero environment with constant-power EIH curing or constant-voltage EIH curing. The CF content was uniformly set at 0.9 vol%. Based on the initial resistance of CF-CM specimens with a CF content of 0.9 vol%, an applied voltage of 13.5 V was determined to ensure the initial electric power of 30 W. Accordingly, the voltage for constant-voltage EIH-cured specimens was set at 13.5 V throughout the entire curing process. The temperature development curves of the CF-CM specimens under different EIH curing regimes are shown in Figure 5. It can be observed that the curing temperature of the specimens developed highly unstably under the constant-voltage EIH curing regime. During the initial curing stage, the specimen temperature rose rapidly. However, as the curing age increased, the curing temperature exhibited a significant downward trend. By 12 h, the curing temperature decreased to below 10 °C, which was insufficient to promote stable performance development of the specimens in the sub-zero environment. Moreover, with further extension of the curing age, the specimen temperature would likely drop below 0 °C, potentially causing a complete cessation of the hydration reaction. In contrast, the curing temperature of specimens subjected to constant-power EIH curing remained stable above 50 °C. This condition is conducive to specimen performance development, and it can be inferred that the subsequent curing temperature would also remain stable. In the following experiments, the constant-power EIH curing regime was employed.

**Figure 6:**
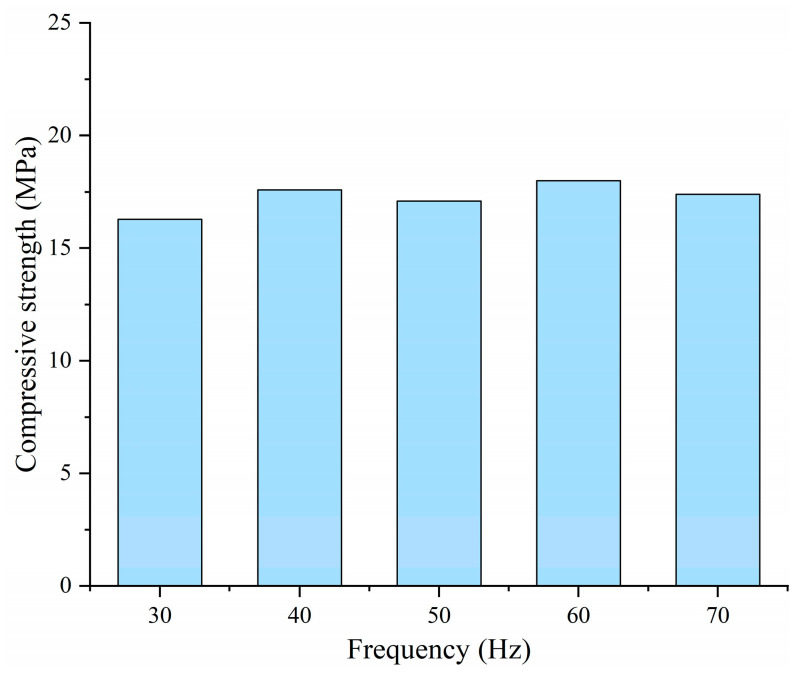
Compressive strengths of EIH-cured CF-CM with different frequencies.

Furthermore, to investigate the influence of applied frequency on the performance of CF-CM subjected to EIH curing, CF-CM specimens with a CF content of 0.9 vol% were cured by EIH curing for 12 h under different applied frequencies (30 Hz, 40 Hz, 50 Hz, 60 Hz, and 70 Hz). During curing, the power applied to the specimens was uniformly maintained at 30 W. As exhibited in Figure 6, the variations in the applied frequency within the tested range did not significantly affect the early-age mechanical strength of EIH-cured specimens, which all fell within the range of 20–25 MPa. These results indicate that the 50 Hz alternating current (AC), commonly used in commercial and industrial mains electricity, is sufficient to ensure the normal development of mechanical strength in EIH-cured specimens. This finding demonstrates the applicability of this method for practical production scenarios.

### 3.4. Effect of CFs on the Mechanical Properties

**Figure 7:**
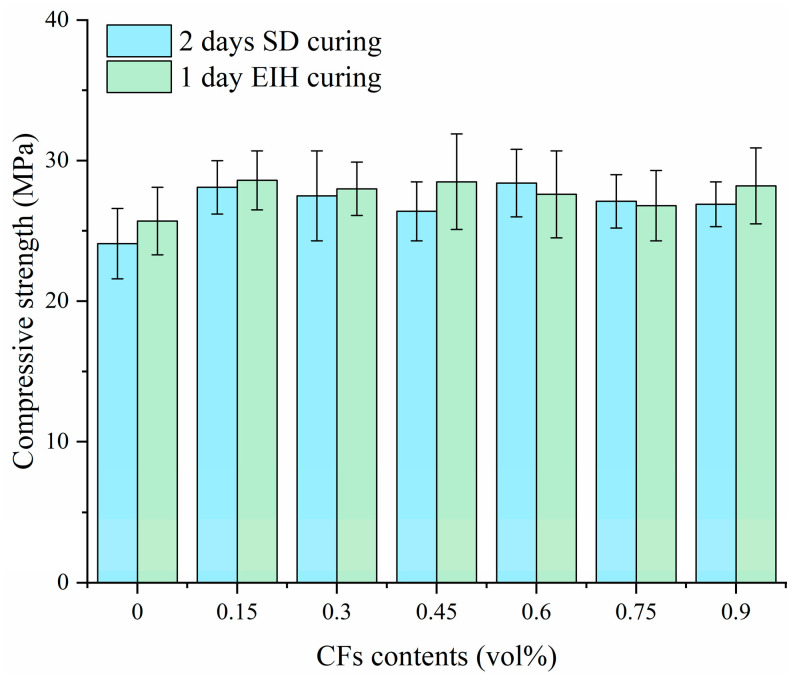
Compressive strengths of CF-CM containing various CF contents.

Figure 7 presents the early-age compressive strength development of CF-CM specimens cured by different curing methods. The results demonstrated that the 1-day EIH curing method achieved compressive strengths comparable to the longer SD curing method under challenging sub-zero condition of −20 °C, highlighting the feasibility of EIH curing for early strength development in cold climates; this equivalence was significant given the harsh −20 °C environment, where conventional hydration processes were severely inhibited. The effectiveness of the short-duration EIH treatment suggested that the applied internal heat during the initial 24 h effectively promoted the strength formation for the specimens to overcome the detrimental effects of low temperature. It can be found that no matter the CF contents inside the specimen, the compressive strengths of EIH specimens were all above 25 MPa. It should be noted that traditional cement concrete failed to gain strength under −20 °C, so the implementation of EIH curing was significant for the winter concrete construction. Moreover, the incorporation of CFs enhanced the compressive strength of the specimen compared to the unreinforced control (0 vol% CFs), which was attributed to the fibers’ role in microcrack bridging, stress distribution, and providing secondary reinforcement. However, the analysis further indicated that increasing the CF content showed little influence on compressive strength. To be specific, the compressive strengths of EIH-cured specimens with varying CF contents were 25.7 MPa, 28.6 MPa, 28.0 MPa, 28.5 MPa, 27.6 MPa, 26.8 MPa, and 28.2 MPa, with the CF contents varying from 0 vol% to 0.9 vol%.

### 3.5. Temperature Distribution Based on Finite Element Analysis

In this work, COMSOL software was used to reflect the temperature distribution in EIH-cured specimen. However, given the inherent complexity of cement-based systems under EIH curing, with evolving hydration reactions, heterogeneous material properties, and dynamic electric–thermal couplings, the modeling process of property evolution remains an outstanding challenge. This work aimed to provide primary electrical–thermal conversion process by finite element analysis, and the more complicated coupled relationship is expected to be disclosed in future work.

As the thermal conductivity of the mortar specimens is related to the CF contents, the thermal conductivities of mortar specimens with different CF contents can be calculated by fitting the polynomial (Equation (1)) based on the relationship curve between thermal conductivity and fiber content. It can be found that the thermal conductivity of mortar decreased with the increase in fiber content.

(1) $$h_{c}=0.9918-0.2741m+0.075m^{2}$$

Here, *h_c_* indicates the thermal conductivity of the specimen, and *m* means the CF content.

**Figure 8:**
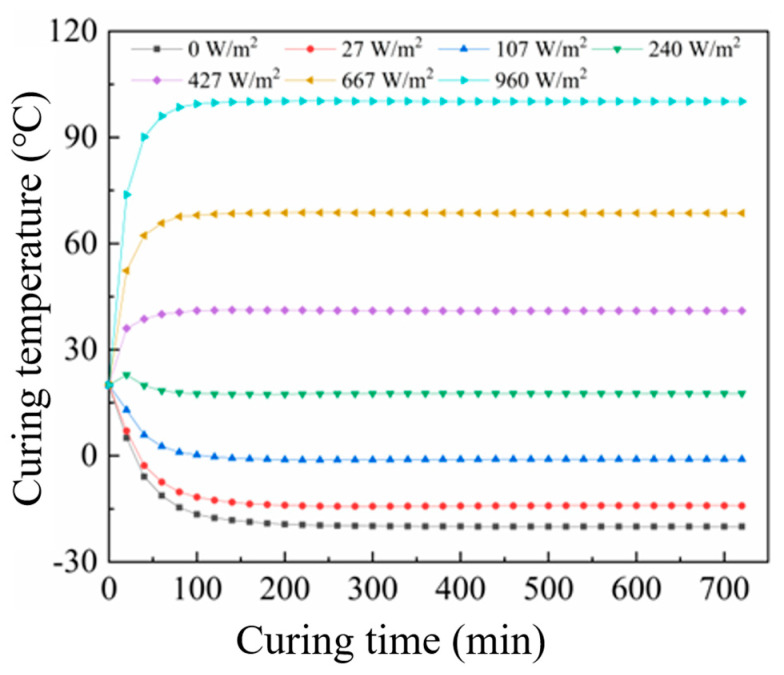
Curing temperature of CF-CM at various electric power densities.

The temperature evolution at the center of CF-CM specimens under different EIH curing power densities, controlled by varying the applied voltage, is shown in Figure 8.

Figure 8 reveals that the stabilized temperature at the center of the specimen increased progressively with higher curing power density. At 0 W/m^2^ (i.e., no electrical input in a −20 °C environment), the center temperature decreased rapidly, falling below −10 °C within 60 min and ultimately equilibrating to the ambient temperature of −20 °C. A power density of 107 W/m^2^ yielded a stabilized center temperature near 0 °C. At 240 W/m^2^, the heat generated by EIH curing balanced the heat loss to the sub-zero environment, stabilizing the center temperature near the initial 20 °C. A power density of 667 W/m^2^ achieved a stabilized center temperature of approximately 65 °C, providing suitable conditions for cement hydration in sub-zero environments and enabling rapid strength development. However, a further increase in the power density to 960 W/m^2^ elevated the stabilized temperature to 100 °C. This excessive temperature may induce detrimental thermal stresses that compromise strength.

**Figure 9:**
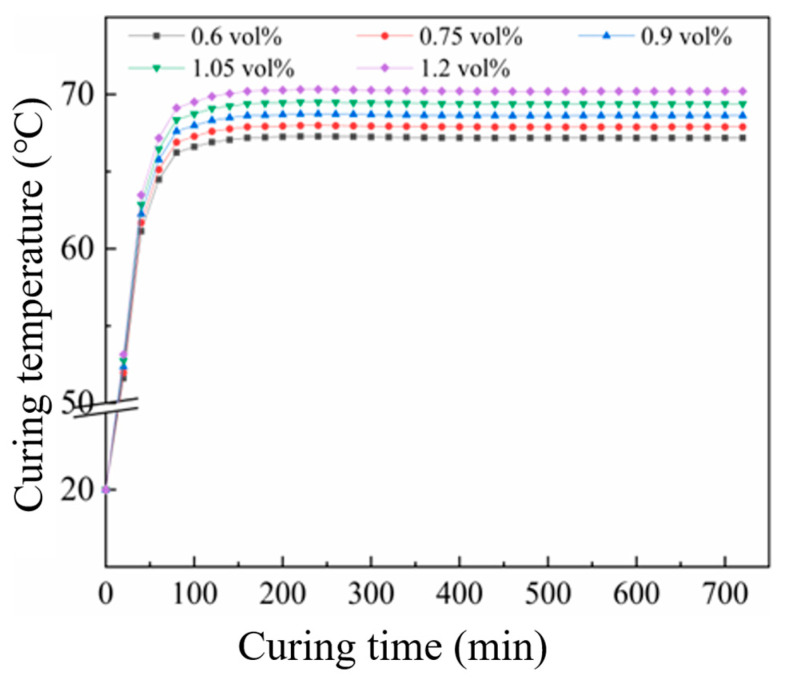
Curing temperature of CF-CM with different CF contents.

Consequently, 667 W/m^2^ was selected as the optimal applied power density for mortar specimens. Parametric scanning of thermal conductivity values was performed to generate temperature–time curves at the center point (as exhibited in Figure 9). It can be seen from the figure that as the CF content increased, the stable temperature at the center point of the specimen model gradually rose. When the CF content was 0.6 vol%, the temperature at the center point of the specimen model finally stabilized at 67.2 °C. When the carbon fiber content reached 1.2 vol%, the temperature at the center point of the specimen model finally remained at 70.2 °C. The final temperatures at the center points of the cement mortar specimen models with various CF contents were all within the suitable curing temperature range (60~70 °C).

**Figure 10:**
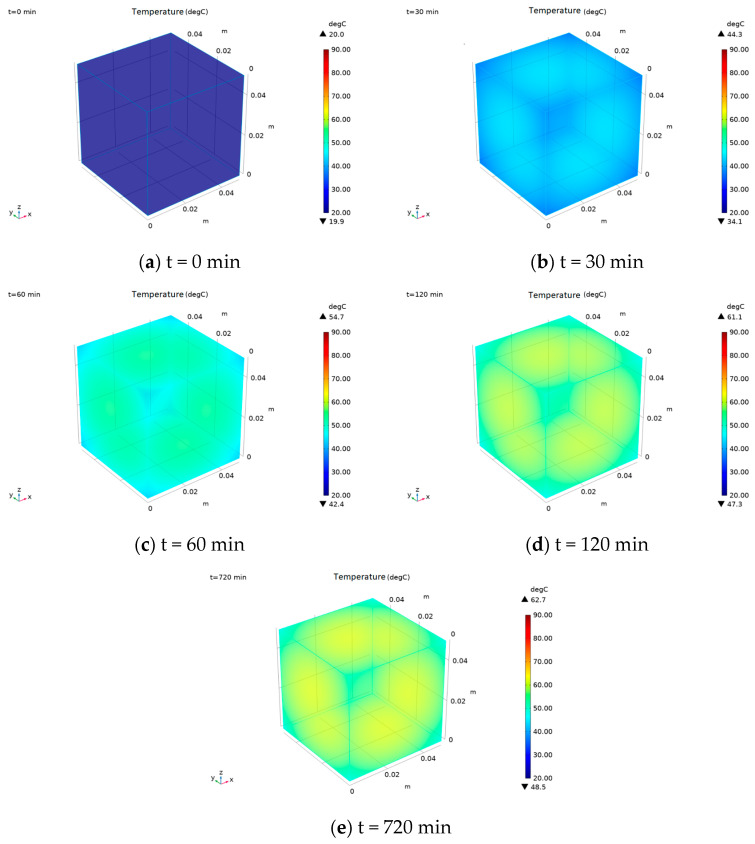
Temperature field of EIH-cured CF-CM specimens at different time points.

Since the center point temperature alone cannot fully characterize the curing temperature distribution of EIH-cured specimens, the temperature field during EIH curing was investigated. In this part, the constant optimal power density (667 W/m^2^) was determined, and the surface temperature distribution of mortar specimens in a −20 °C environment was simulated at various curing times (Figure 10). The results showed a slightly elevated temperature at the surface center, decreasing radially towards the edges. The four corners exhibited the lowest surface temperatures due to their maximum interfacial area with the environment. To be specific, the bigger interfacial area of the specimen between environment can facilitate greater heat dissipation, which can influence the curing temperature distribution of EIH-cured specimens. Moreover, during the first 60 min of EIH curing, the overall surface temperature increased continuously. However, the corner regions warmed slower than the central areas, and this temperature differential widened with prolonged curing. Beyond 120 min, the temperature stabilized uniformly across most of the specimen surface.

## 4. Conclusions and Prospect

In this work, EIH curing was used to prepare CF-CM in a sub-zero environment of −20 °C. A series of experiments were conducted to clarify the factors influencing the performance of EIH-cured CF-CM. Furthermore, FEM analysis was conducted for a better understanding in the electrical–thermal conversion during EIH curing process. The main conclusions can be summarized as below.

(1) The electrical percolation threshold of CFs within the CF-CM specimen was experimentally determined to be 0.6 vol% based on the variation curve of initial electrical resistivity with different CF contents. Compressive strength test results revealed that 1 day of EIH curing can effectively promote the strength formation of CF-CM under −20 °C, which was comparable to that of specimens subjected to SD curing for 2 days. It was also observed that the change in CF contents showed limited influence on the strength of CF-CM.

(2) The preparation method greatly affected the compressive strength of CF-CM. Method IV endowed the specimen with the highest strength, which may be attributed to better CF distribution. Constant-power EIH curing kept temperatures stable above 50 °C. Changing the electrical frequency (30–70 Hz) during EIH curing had almost no effect on early strength, with all samples showing similar compressive strength results. This proves that standard 50 Hz AC power works perfectly for EIH curing, making it practical for real-world use.

(3) COMSOL simulations confirmed uniform surface heating within 120 min, though corners remained cooler due to greater heat dissipation. This study established 667 W/m^2^ as the optimal power density for EIH curing of CF-CM at −20 °C, maintaining stable core temperatures of 60–70 °C for rapid strength development. Higher CF content (0.6–1.2 vol%) increased thermal conductivity but reduced overall thermal conductivity. Crucially, exceeding 960 W/m^2^ caused damaging temperatures (>100 °C), while insufficient power (107–240 W/m^2^) failed to sustain hydration temperatures in sub-zero environments.

Collectively, this work presents EIH curing as an efficient method for the strength formation of cement mortar under −20 °C, and the effects of some crucial parameters were clarified. However, it should also be emphasized that only early-age performance of EIH-cured specimen was elucidated in this work, and the more complicated couple mechanisms among different reactions in EIH-cured specimen were not included in the simulation process. The researchers are encouraged to conduct deeper research regarding the above issues.

## Figures and Tables

**Table 1:** Chemical composition of cement (wt%).

Chemical Composition	CaO	SiO_2_	Al_2_O_3_	Fe_2_O_3_	SO_3_	MgO
Content (wt%)	63.15	20.87	5.38	3.64	2.33	1.64

**Table 2:** Mineralogical composition of cement (wt%).

C_3_S	C_2_S	C_3_A	C_4_AF
57.23	18.01	7.21	11.34

**Table 3:** Preparation methods for CF-CM.

	Addition Sequence	Mixing Methods
Method I	First, the CFs and cement were added, followed by the addition of water, and the sand was finally incorporated.	The CFs and cement were slowly stirred for 3 min; after adding water, the mixture was further slowly stirred for 3 min followed by another 3 min of mixing after adding sand.
Method II	First, the CFs, cement, and sand were added, and then the water was incorporated.	The CFs, cement. and sand were stirred slowly for 3 min; after adding water, the mixture was slowly stirred for 3 min, followed by 3 min of quick stirring.
Method III	First, the CFs, cement, and sand were added, and then the water was incorporated.	The CFs, cement, and sand were stirred slowly for 3 min; after adding water, the mixture was quickly stirred for 3 min, followed by 3 min of slow stirring.
Method IV	First, the CFs, cement, and sand were added, and then the water was incorporated.	The CFs, cement, and sand were stirred slowly for 2 min, followed by 3 min of quick stirring; after adding water, the mixture was quickly stirred for 3 min.

## Data Availability

The original contributions presented in the study are included in the article; further inquiries can be directed to the corresponding author.
